# Ecology of phlebotomine sand flies in a Brazilian area with recent leishmaniasis transmission (Itaúna, in Minas Gerais state)

**DOI:** 10.1590/0037-8682-2019-0538-2019

**Published:** 2020-04-03

**Authors:** Nathália Cristina Lima Pereira, Érika Monteiro Michalsky, Fabiana Oliveira Lara-Silva, Rosana Silva Lana, Adão Júnior Viana de Paula, Daniele Marques Pereira, Josiane Valadão Lopes, Consuelo Latorre Fortes-Dias, Edelberto Santos Dias

**Affiliations:** 1 Instituto René Rachou, Fundação Oswaldo Cruz, Belo Horizonte, MG, Brasil.; 2 Fundação Ezequiel Dias, Belo Horizonte, MG, Brasil.

**Keywords:** Leishmaniasis, Phlebotominae, Synanthropy index, Lutzomyia longipalpis, Nyssomyia whitmani

## Abstract

**INTRODUCTION:**

Leishmaniasis constitutes a serious but neglected tropical disease. Recently, socio-environmental, biological and physical changes have altered the range of leishmaniasis, causing it to spread into urban areas. In Minas Gerais, the disease is endemic, exhibiting regional differences and reaching urban centers. The purpose of this study was to evaluate entomological aspects related to the ecoepidemiology of leishmaniasis in Itaúna.

**METHODS:**

Monthly catches with HP traps were carried out from June 2017 to May 2018, in three ecological areas (urban, rural, and forest). The adaptability of the species to anthropic environments was assessed using the synanthropy index (SI).

**RESULTS:**

We collected 1306 specimens of phlebotomine sand flies. Of the species of medical importance, *Lutzomyia longipalpis,* the vector of *Leishmania infantum,* represented 90.4% of the specimens identified at species level (n=1260). *Nyssomyia whitmani*, the vector of *Leishmania braziliensis*, represented 1.6% of the total. *Lu. longipalpis* displayed an SI of +95.8, a value that denotes a marked preference for human environments. For *Ny. whitmani*, the SI was -25, expressing the tendency of this species to occupy uninhabited areas. The population of the three most numerous species captured increased with rain, high temperatures, and high relative humidity. Although captured at low numbers, *Ny. whitmani* showed a different profile concerning the climate variables analyzed.

**CONCLUSIONS:**

Understanding the epidemiology of the disease may assist the health services in formulating effective control measures for improving community health and contributing to the establishment of a dynamic relationship and a global awareness of the health/disease process.

## INTRODUCTION

Leishmaniases are anthropozoonotic infectious diseases transmitted by the bite of *Leishmania*-infected female phlebotomine sand flies. In the American continent, their two basic forms are visceral leishmaniasis (VL) and cutaneous leishmaniasis (CL)^1^. Previously, leishmaniases were associated with rural and non-anthropic areas, where human beings could accidentally get infected by passing through or colonizing infected areas^2^. Over the years, this pattern has changed, and currently the transmission cycle also occurs in urban centers^3^.

Epidemiological studies have shown that changes in the transmission profile and increasing urbanization of leishmaniases can be due to deforestation and the haphazard growth of cities, which reduces and modifies the natural habitats of these insects, restricting their environment and causing species adaptation^4^ ^,^ ^5^. Nuorteva^6^ named the ability of certain species to adjust to human-created or modified environmental conditions, which generally result from urbanization, synanthropy. The synanthropy index (SI) measures the degree of adaptability of species to urbanized environments and has been studied from an ecological perspective to evaluate the results of human influence on the original fauna of an environment.

Studies have shown that climatic factors, such as temperature, rainfall and relative humidity can also correlate with the occurrence of phlebotomine sand flies and their population density^7^. An understanding of the ecology of *Leishmania* vectors and their monthly distribution associated with these climate variables can contribute to a better comprehension of the epidemiology of leishmaniases and their dynamics and possibly improve the effectiveness of control strategies in transmission areas^8^.

The Ministry of Health (MS) has recently classified Itaúna (Minas Gerais) as an area of sporadic transmission of VL. The municipality is concerned because of the increased number of human and canine cases, the presence of the competent vector (*Lu. longipalpis*) and Itaúna’s proximity to endemic areas. Thus, in order to elucidate the ecology of the phlebotomine sand flies in relation to the epidemiology of leishmaniasis, we started a project in the municipality of Itaúna. This involved a survey of the sand fly fauna, calculation of the synanthropic index of each species captured and evaluation of the influence of climate on the population density of these insects. These data may contribute to the establishment of a dynamic relationship and a global understanding of the health/disease process, leading to more effective and integrated measures for efficient disease control in Itaúna.

## METHODS

### Study Area

This study was conducted in Itaúna (20°4ʹ26″S, 44°34ʹ24″W), a municipality in the state of Minas Gerais, Brazil. Itaúna is located in the midwestern region of the state, 76 km from Belo Horizonte. According to the Brazilian Institute of Geography and Statistics^9^ the municipality comprises an area of 495,769 km², with approximately 72 neighborhoods and an estimated population of 92,561 inhabitants. The region is hilly with a clayey soil and a tropical climate with hot summers and dry winters. Average altitude is 893 m with minimum of 711 m and maximum of 1321 m. Itauna’s biome is cerrado and Atlantic Forest^9^.

### Capture and Identification of Phlebotomine Sand Flies

The entomological captures were made in three ecologically distinct areas: urban, rural, and forest. The favorable criteria considered for the selection of capture points were the presence of fruit trees, abundant vegetation, accumulation of organic matter in the soil, and presence of chicken-coops and domestic animals. Thus, the urban site selected for this study was a residence located in a neighborhood called "Chácara do Quitão" (20°05ʹ32″S, 44°35ʹ55″W, altitude 864 m), which is predominantly residential and has a dense human occupation, located near the city center, with the presence of chickens, pigs and dogs. The rural area selected for this study was a farm in Piaguassu neighborhood (20°03ʹ26″S, 44°36ʹ55″W, altitude 818 m), with few dwellings and the presence of pasture, gardens, chickens, pigs, cattle, goats, dogs, and cats. The selected forest area belongs to the Zoonoses Control Center (ZCC), located in the Industrial District (20°02ʹ26″S, 44°36ʹ54″W, altitude 844 m), at a distance of 10 km from the urban center of Itaúna, which has a vast area of transitional forest (cerrado and semi-deciduous seasonal forest) with wild birds and mammals, a small perennial stream, and low solar incidence. In the forest area, the ZCC was taken as reference for placement of the traps. To minimize any possible bias due to sporadic and intermittent presence of horses and dogs in ZCC facility, the traps were mounted at about 300 m from the facility.

The captures were performed monthly, during three consecutive nights, from June 2017 to May 2018, using three HP light traps^10^ for each ecological area. In each area, the traps were placed at 300 m from each other. The male specimens were stored in 70% alcohol for subsequent taxonomic identification, and the females were conditioned in 6% DMSO and stored at -20° C for further dissection (taxonomic identification). Identification at species level was performed according to the classification proposed by Galati^11^ with abbreviation of the generic names as proposed by Marcondes^12^.

### Climate data

Monthly climate data, including temperature (°C), relative humidity (%), wind speed (m/s), and rainfall (mm³), were obtained from the nearest weather station in the municipality (OMM83635), District of the Brazilian Institute of Meteorology of Divinópolis, through the National Institute of Meteorology^13^. Divinópolis is located approximately 43 km from Itaúna (20°8ʹ21″S 44°53ʹ17″W). 

### Synanthropy index

The synanthropy index (SI) was calculated according to Nuorteva formula^6^. The index ranges from +100 (maximum) to -100 (minimum), with positive values representing the highest degree of association with man and negative values indicating aversion to the urban environment.

$$SI=\;\frac{2a+b-2c}{2}$$

where

*a* = percentage of a species captured in urban areas

*b* = percentage of the same species captured in rural areas 

*c* = percentage of the same species captured in forest areas

## RESULTS

During the 12 months of the study period, 1306 phlebotomine sand flies were captured, among which 1260 were identified at level species. The sand flies captured belonged to six genera (*Lutzomyia, Evandromyia*, *Pintomyia*, *Nyssomyia*, *Psathyromyia* and *Brumptomyia*), and included 11 species: *Brumptomyia brumpti* (Larrousse, 1920), *Evandromyia cortelezzii* (Brèthes, 1923), *Evandromyia evandroi* (Costa Lima & Antunes, 1936), *Evandromyia lenti* (Mangabeira, 1938), *Evandromyia sallesi* (Galvão & Coutinho, 1940), *Evandromyia termitophila* (Martins, Falcão & Silva, 1964), *Lu. longipalpis* (Lutz & Neiva, 1912), *Nyssomyia whitmani* (Antunes & Coutinho, 1939), *Pintomyia pessoai* (Coutinho & Barreto, 1940), *Psathyromyia brasiliensis* (Costa Lima, 1932), and *Psathyromyia lutziana* (Costa Lima, 1932) (Table 1). Among them, 1099 were males (87.5%) and 161 females (12.5%), with an M/F ratio of 7.0. Thirty-eight females were identified as belonging to the *cortelezzii* complex because female specimens of *Ev. cortellezzii* and *Ev. sallesi* are indistinguishable. Forty-six specimens were considered unidentified (3.5%) because the morphological characters necessary for their identification at species level were missing. The main species of medical importance were *Lu. longipalpis* (VL vector), which represented 90.4% of the specimens captured, and *Ny. whitmani* (CL vector), 1.6%.

**TABLE 1:** Distribution of male and female of phlebotomine sand fly species (n = 1260) captured in Itaúna, Minas Gerais state, Brazil. June 2017 to May 2018.

Species	Urban area			Rural area			Forest area			Total			
	♂	♀	Both genders	♂	♀	Both genders	♂	♀	Both genders	♂	♀	Both genders	%
*Br. brumpti*	1	2	3	-	1	1	-	1	1	1	4	5	0.40
*cortelezzii complex*	-	28	28	-	6	6	-	4	4	-	38	38	3.02
*Ev. cortelezzii*	5	-	5	-	-	-	-	-	-	5	-	5	0.40
*Ev. evandroi*	5	5	10	-	-	-	-	-	-	5	5	10	0.79
*Ev. lenti*	8	13	21	-	5	-	-	1	1	8	19	27	2.14
*Ev. sallesi*	1	-	1	-	-	-	-	-	-	1	-	1	0.08
*Ev. termitophila*	-	1	1	-	1	1	1	-	1	1	2	3	0.24
*Lu. longipalpis*	977	73	1050	84	3	87	1	1	2	1062	77	1139	90.40
*Ny. whitmani*	2	1	3	3	3	6	6	5	11	11	9	20	1.59
*Pi. pessoai*	-	-	-	3	-	3	1	4	5	4	4	8	0.63
*Pa. brasiliensis*	-	-	-	-	-	-	-	1	1	-	1	1	0.08
*Pa. lutziana*	1	-	-	-	-	-	-	2	2	1	2	3	0.24
**Total**	**1000**	**123**	**1023**	**90**	**19**	**109**	9	**19**	**28**	**1099**	**161**	**1260**	**100.00**

**FIGURE 1:**
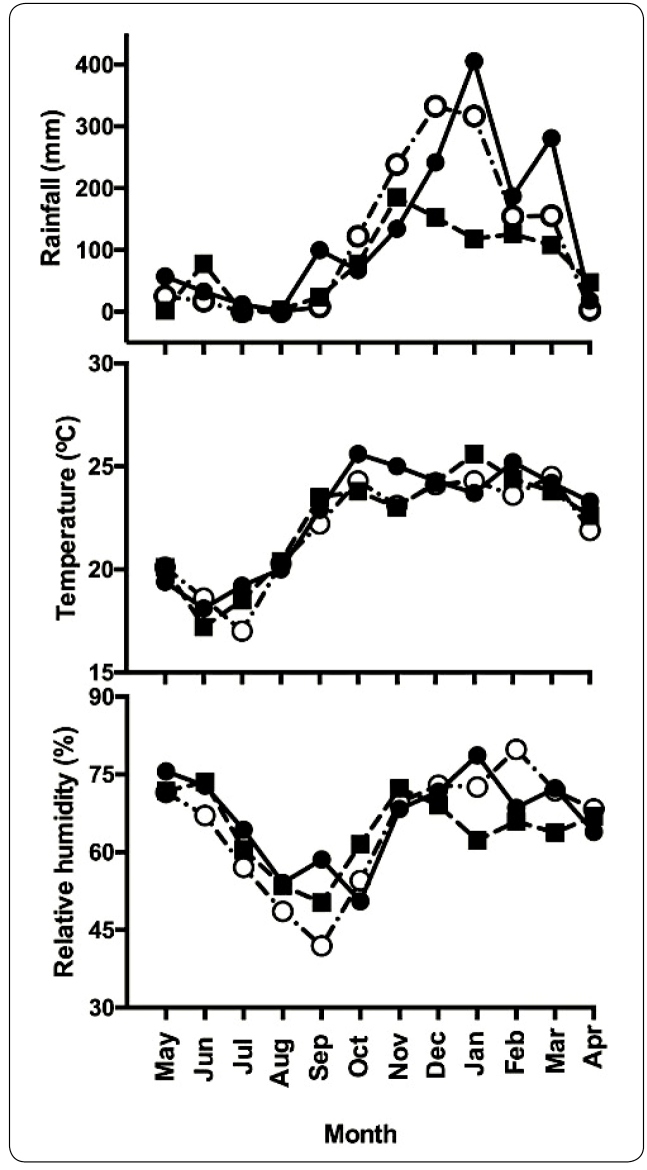
Historical series of main climate variables (rainfall, temperature and relative humidity) in Itaúna, Minas Gerais state, Brazil. Legend: black circle - 2015/2016, black square 2016/2017; white circle - 2017/2018 (present study).

**FIGURE 2:**
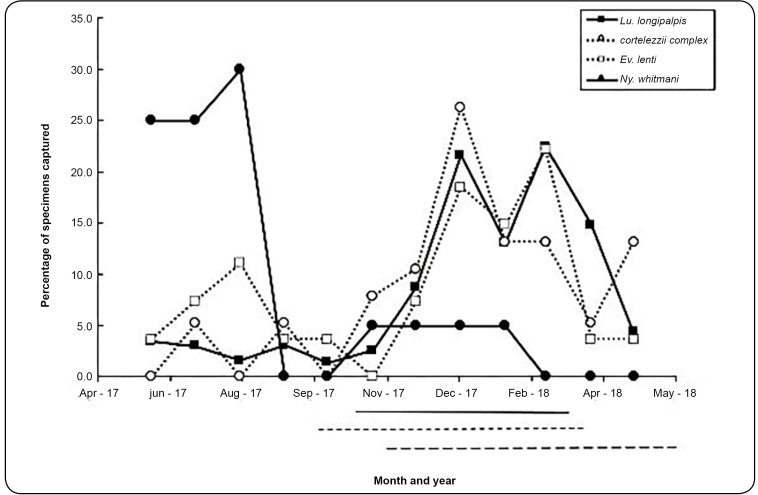
Population curves of the four most numerous species of phlebotomine sand flies captured in Itaúna, Minas Gerais state, Brazil. Months with climate variables above the averages in the studied period are marked by lines below the abscissa: rainfall - continuous line; temperature - short dashed line; relative humidity- long dashed line. June 2017 to May 2018.

During the period studied, monthly rainfall ranged from 0 mm to 332.5 mm. The average temperature and relative humidity were 21.9 (standard deviation [SD] 2.6 °C and 64.3 (SD 11.3%), respectively. The dry season extended from Apr. to Sept. and the rainy season from Oct. to Mar. The overall profile of the three main climate variables was similar to that of the previous two years (Figure 1). The population curve of the predominant species in our study (*Lu. longipalpis*) is shown in Figure 2. A populational peak was observed in the warm and rainy season, when relative humidity increases. Although captured at much smaller numbers compared to *Lu. longipalpis* (Table 1), population curves were also constructed for the *cortelezzii* complex, *Ev. lenti*, and *Ny. whitmani*. Whereas populations of the first two species oscillated in a similar way to *Lu. longipalpis*, *Ny. whitmani* showed a populational peak in the dry season. 

**FIGURE 3:**
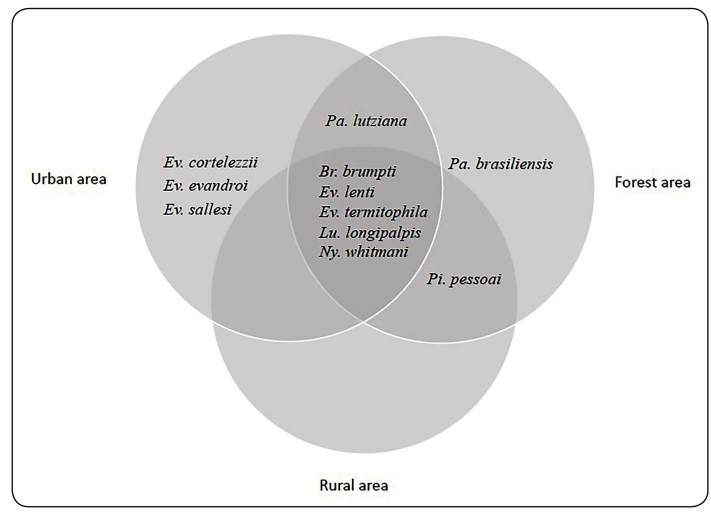
Distribution of phlebotomine sand fly species in forest, rural, and urban areas. Itaúna, Minas Gerais, Brazil. June 2017 to May 2018

Throughout the study period, 1123 (89.1%) specimens were captured in the urban area, 109 (9.7%) in the rural area, and 28 (2.2%) in the forest. *Ev. cortelezzii*, *Ev. evandroi* and *Ev. sallesi* were caught only in the urban area, and *Pa. brasiliensis* only in the forest. The other species were found in at least two of the three ecological areas, in various proportions. Regarding species richness, nine species were captured in the urban area, six species in the rural area and eight species in the forest. These results are represented by a logic diagram (Figure 3). A greater number of males was captured in urban and rural areas, while a higher number of females was captured in the forest, all of them identified at species level (Table 1).

Regarding the synanthropy index, *Ev. evandroi* showed absolute synanthropy (SI +100) and *Pa. brasiliensis* showed absolute asynanthropy (SI -100). *Ev. lenti* and *Lu. longipalpis* showed a high degree of adaptability to the urban environment, with SI +83.4 and SI +95.8, respectively (Table 2). Considering that the *cortelezzii* complex included only females and that *Ev. cortelezzi* and *Ev. sallesi* were only represented by male specimens, it was not possible to calculate their synanthropy index.

**TABLE 2:** Synanthropy index (SI) for phlebotomine sand fly species captured in three areas (forest, rural and urban) of Itaúna, Minas Gerais state, Brazil, from June 2017 to May 2018.

Species	SI
*Br. brumpti*	+50.0
*Ev. evandroi*	+100.0
*Ev. lenti*	+83.4
*Ev. termitophila*	+16.6
*Lu. longipalpis*	+95.8
*Ny. whitmani*	-25.0
*Pi. pessoai*	+95.8
*Pa. brasiliensis*	-100.0
*Pa. lutziana*	-33.4

## DISCUSSION

The geographical distribution of leishmaniases in the country indicates that its epidemiology is influenced by a great range of environmental, climatic and socioeconomic aspects^7^ ^,^ ^14^ ^-^ ^16^. Moreover, the emergence of new risk factors has favored human-vector contact, leading to new epidemiological scenarios and increasing the incidence of leishmaniases. These new epidemiological scenarios might demand improvements in the control program^17^ ^-^ ^19^.

Over recent years, Minas Gerais has experienced a significant increase in the number of reported cases of VL, representing 71% of the total cases recorded in the southeast of Brazil^20^. During our study in the municipality of Itaúna in Minas Gerais state, 1306 specimens were captured, with a fauna of 11 species, distributed in six genera (Table 1). Among the species, we highlight the presence of *Lu. longipalpis* and *Ny. whitman*i, which are acknowledged vectors of *Le. infantum* and *Le. braziliensis*. These results are similar to those found in another study conducted in the municipality, where the same genera and almost all the same species were captured^21^.

As climatic factors may influence the population dynamics of phlebotomine sand flies, it is fundamentally important to understand the seasonal distribution of these insects for the implementation of effective vector control programs^22^. Climate variables have a definite impact on phlebotomine populations, depending on the region. By analyzing the relationship between climate data and population fluctuation in *Lu. longipalpis,* our results demonstrate peaks of capture in warm and wet months (with higher relative humidity and rainfall) in Itaúna (Figure 2). Other studies performed in Brazil also found peaks of *Lu. longipalpis* during hot and rainy months^21^ ^,^ ^23^ ^,^ ^24^ ^,^ ^26^ ^-^ ^29^. The distinct profile found for *Ny. whitmani* may be regarded with caution due to the small number of specimens (20 specimens) captured in our study (Figure 2). 

The occurrence of a particular sand fly species in a given ecological area (urban, rural or forest) is related to its adaptation to environmental conditions, resource availabilities and its competitive interaction with other species^30^. In the present study, the richness and abundance of species varied between the localities, confirming that environmental or geographical factors may determine a different configuration of phlebotomine sand fly populations. When analyzing the density of sand fly species in each ecological area, it was observed that the smallest number of specimens captured occurred in the forest area (2.1%) (Figure 3). Studies carried out in forest environments showed a phlebotomine fauna generally composed of a few dominant species and many species with few specimens, corroborating our findings^31^ ^,^ ^32^. In contrast, the largest number of captured specimens was found in the urban area (89.3%) (Figure 3), and this may be due to the presence of kennels, chicken-coops and fruit trees, which present ideal characteristics for the proliferation of sand flies^33^ ^,^ ^34^. Some authors highlight the relevance of animal shelters as places for establishment and maintenance of a high sand fly density in the environment and consider these places as risk factors^11^ ^,^ ^35^ ^,^ ^36^. However, the rural area also presents such characteristics and a considerably smaller number of specimens was captured here when compared to the urban area (8.58%). This fact indicates the importance of studying the fauna of different ecological areas and the behavior of each species captured.

In the present study, a high density of the predominant species (*Lu. longipalpis*) was found in the urban environment (92.2%) with SI +95.8 (Table 2). The abundance and frequency of this species in the urban area of Itaúna resemble those recorded by several other studies in Brazil^37^ ^-^ ^41^, including a recent entomological fauna survey developed by our group in Itaúna^21^. According to Melo^42^, among all sand fly species, the most adapted to the anthropic environment is *Lu. longipalpis*. Its high degree of adaptation to the peridomestic environment of anthropic areas is mainly influenced by the presence of domestic animals^21^ ^,^ ^24^ ^,^ ^25^,^26^ ^,^ ^27^ ^,^ ^40^ ^,^ ^43^. In the forest area, ZCC was taken as reference for the placement of traps. The low number of *Lu. longipalpis* captured in the area (two specimens only) indicated that the distance between the traps and ZCC was adequate. The sporadic presence of horses and dogs in the ZCC did not bias the results. 

Some sand fly species may be in the early stages of adaptation to the urban environment and, although apparently rare and without epidemiological importance, they would be adaptable and able to perform vector functions^44^. Despite previous studies on sand fly biology and behavior, some gaps related to the synanthropy of these insects remain to be clarified.

Our results emphasize the importance of studying the ecology/biology and behavior of phlebotomine sand flies, demonstrating their epidemiological role in the transmission of leishmaniases in urban centers. Thus, the potential of some species of phlebotomine sand flies as vectors of *Leishmania*, along with their habits and synanthropy, should be considered by the competent health agencies.
